# The effects of fenugreek (*Trigonella foenum-graecum*) seed on glycemic parameters: An updated systematic review and meta-analysis of randomized controlled trials

**DOI:** 10.22038/ajp.2025.26043

**Published:** 2025

**Authors:** Fatemeh Chehregosha, Laleh Fakhr, Ali Tarighat-Esfanjani, Leila Maghsoumi-Norouzabad

**Affiliations:** 1 *Research Center for Integrative Medicine in Aging, Aging Research Institute, Tabriz University of Medical Sciences, Tabriz, Iran *; 2 *Department of Clinical Nutrition, Faculty of Nutrition and Food Sciences, Tabriz University of Medical Sciences, Tabriz, Iran *; 3 *Student Research Committee, Tabriz University of Medical Sciences, Tabriz, Iran*; 4 *Traditional Medicine and Hydrotherapy Research Center, Ardabil University of Medical Sciences, Ardabil, Iran*

**Keywords:** Fenugreek, Fasting blood glucose, Hemoglobin A1c, HOMA-IR, 2hr postprandial glucose, Insulin

## Abstract

**Objective:**

The presented meta-analysis of randomized controlled trials aimed to analyze the effectiveess of fenugreek (*Trigonella foenum-graecum*) on fasting blood glucose (FBG), 2-hr postprandial glucose (2hPPG), Hemoglobin A1c (HbA1c), Insulin and Insulin resistance (HOMA-IR).

**Materials and Methods:**

A systematic literature search of several databases was performed from inception to 30 October 2023, for controlled clinical trials. Data were analyzed using the random-effect model, and are presented as weighted (WMD) or standardized (SMD) mean difference and associated 95 % confidence interval (CI). Heterogeneity between studies was assessed using the Cochrane χ^2^ test. Meta-regression, subgroup analysis, and sensitivity analysis were used to identify the source of heterogeneity. Funnel plot, Egger's, and Begg's tests were also used to evaluate publication bias.

**Results:**

A total of 26 Randomized controlled trial (RCTs) met the eligibility criteria. The results indicated significant improving effects of fenugreek on FBG (WMD: − 16.75 mg/dl; 95 % CI: − 23.36, − 10.15; p<0.001), 2hPP (WMD: - 22.28 mg/dl; 95 % CI: - 34.42 to - 10.15; p<0.001; I² (%): 95.1%, p<0.001), HbA1c levels (WMD: - 0.63 mg/dl; 95 % CI: - 0.76 to - 0.51; p*<*0.001), and insulin (SMD: - 0.42; 95 % CI: - 0.79 to - 0.05; p = 0.026). However, the HOMA-IR effect was insignificant (WMD: -22.28 mg/dl; 95 % CI: - 0.84 to 0.02; p = 0.061).

**Conclusion:**

The overall results support the possible protective and therapeutic effects of fenugreek on glycemic parameters. Future studies with higher quality are necessary to confirm the results of the present meta-analyses.

## Introduction

Type 2 diabetes mellitus (T2DM) is one of the most common chronic diseases and a leading cause of death worldwide, affecting individuals across all countries, different age groups, and both genders. Insulin resistance and beta-cell dysfunction are the two main contributors to T2DM which is characterized by chronic hyperglycemia (Chan et al. 2020; Jackson-Morris et al. 2023). According to the International Diabetes Federation (IDF), in 2021, the global population of individuals living with diabetes was 529 million, with an age-standardized total diabetes prevalence of 6.1%. The prevalence of diabetes is projected to reach 1.31 billion people worldwide by 2050 (Kanyin Liane Ong 2023). 

The continuous increase in blood glucose levels in uncontrolled diabetes can lead to various acute and chronic complications. Diabetes mellitus is one of the primary causes of cardiovascular diseases (CVD), blindness, kidney failure, and lower limb amputation (Chiong and Evans-Molina 2013). Lifestyle changes, including weight loss, regular physical activity, a healthy diet, and consistent use of medications, are the first line of treatment for diabetes management (Franz et al. 2015). However, due to the side effects associated with medications, herbal remedies have received renewed attention in recent years. In this context, fenugreek, also known as *Trigonella foenum-graecum* of the Fabaceae family, is considered one of the most common herbal medicines. It contains active components such as soluble fiber and a variety of phytochemicals including saponins, trigonelline, diosgenin, and 4-hydroxyisoleucine (Hosseini et al. 2023; Nagulapalli Venkata et al. 2017; Zandi et al. 2015), with various therapeutic properties. It is widely used, particularly in countries such as China, India, Iran, and Egypt, for cooking and as a complementary therapy for diabetes mellitus. Additionally, it plays a role in preventing and improving the complications associated with diabetes (Gong et al. 2016; Hashempur et al. 2015; Shabil et al. 2023; Wang and Wylie-Rosett 2008). To date, several studies have investigated the effects of consuming different forms of fenugreek on glycemic status and hyperinsulinemia. The results of these studies have demonstrated that the consumption of fenugreek seeds can increase insulin sensitivity (Ranade and Mudgalkar 2017) and decrease the levels of insulin and postprandial glucose (Alamdari, Choobineh and Jadidi 2009; Kassaian et al. 2009). Several long-term trials involving fenugreek have also demonstrated a reduction in fasting blood glucose (FBG), 2-hr postprandial glucose (2hPPG), and Hemoglobin A1c (HbA1c) (Gupta, Gupta and Lal 2001; Lu et al. 2008); however, some studies have shown no significant effect (Chevassus et al. 2010; Mathern et al. 2009). Therefore, its effectiveness for glycemic control has not yet been fully confirmed, and there is a need for more clinical studies. However, recent meta-analyses have demonstrated significant effects on certain glycemic parameters, although these studies did not assess insulin and HOMA-IR (Khodamoradi et al. 2020; Shabil et al. 2023). Therefore, the present comprehensive meta-analysis was conducted to evaluate the effect of fenugreek seed on glycemic parameters including FBG, 2hPPG, HbA1c, as well as Insulin and Insulin resistance (HOMA-IR)**.**

## Materials and Methods

The PRISMA-P guidelines, which are the preferred reporting items for systematic reviews and meta-analyses, were followed when conducting this investigation (Page et al. 2021). The review was performed using the PICO design (Uman 2011): Population (adult individuals), intervention (fenugreek consumption), comparison (matched control group), and outcomes (FBG, 2hPP Glucose, HbA1c, Insulin, and HOMA-IR)) applied to the present systematic review and meta-analysis of randomized controlled trials. In summary, the search strategy was as follows:

**Search strategy**

Embase, PubMed, Web of Science, Scopus, and Google Scholar electronic databases were searched from inception to 30 October 2023 using the following search terms and keywords ("Trigonella"[MeSH Terms] OR ("Fenugreek"[Text Word] OR "Fenugreeks"[Text Word] OR "Foenumgraecum"[Text Word] OR "foenum"[Text Word] OR "graecum"[Text Word])) AND “fasting blood glucose” OR glucose OR insulin OR “glycosylated hemoglobin” OR HbA1C OR FBG OR fasting blood glucose OR FBG OR quantitative insulin sensitivity check index OR QUICKI OR Hemoglobin A1c OR HbA1c OR assessment-estimated β-cell function OR homeostasis model of assessment-estimated insulin resistance OR HOMA-IR AND ("intervention"[Text Word] OR "controlled trial"[Text Word] OR "randomized clinical trial"[Text Word] OR "randomized controlled trial"[Text Word] OR "trial"[Text Word] OR "clinical trial"[Text Word] OR "randomized"[Text Word] OR "random"[Text Word] OR "randomly"[Text Word] OR "placebo"[Text Word] OR "RCT"[Text Word]). The search was restricted to English-language articles only. We manually searched the reference lists of all qualifying research to see whether any eligible articles were missing. The study protocol was registered in PROSPERO (Prospective Register of Systematic Reviews) (CRD42023459197) and was approved by the Ethics Committee of Tabriz University of Medical Sciences, Tabriz, Iran (Ethics code; IR.TBZMED.REC.1402.678).

**Study selection**

**Inclusion criteria**

This systematic review included studies that met the following criteria: (a) Clinical trials that are parallel or cross-over; (b) an analysis of how fenugreek affects any of the glycemic parameters in people over the age of eighteen; (c) any study that provided FBG, glucose, insulin, or Hemoglobin A1C levels at baseline and the end of follow-up in each group or presented the net change values. 

**Exclusion criteria**

We excluded studies if they were: (a) uncontrolled trials; (b) conducted on children, pregnant women, animals, or involving *in vitro* studies; (c) had an intervention duration of less than one week with fenugreek; (d) studies that involved other interventions alongside fenugreek; (e) studies that included fenugreek ingredients; or (f) review studies. 

**Data extraction**

First, the titles and/or abstracts were independently assessed by F.C. and L.F. Then, F.C., L.M., and L.F. reviewed the titles and/or abstracts one by one as the initial step. In the second step, the full texts of the remaining publications were obtained. The following data were extracted by the same reviewers: (a) study characteristics (first author, publication date, country of origin, study design, and overall sample size); (b) characteristics of participants (gender, mean age, mean body mass index (BMI), and health status); (c) data on the intervention and comparison (form of fenugreek, dosage, and duration of study); and (d) outcome measures (mean and standard deviation of changes in FBG, 2hPP, HbA1c, insulin, and HOMA-IR.

**Quality assessment**

**Figure 1:**
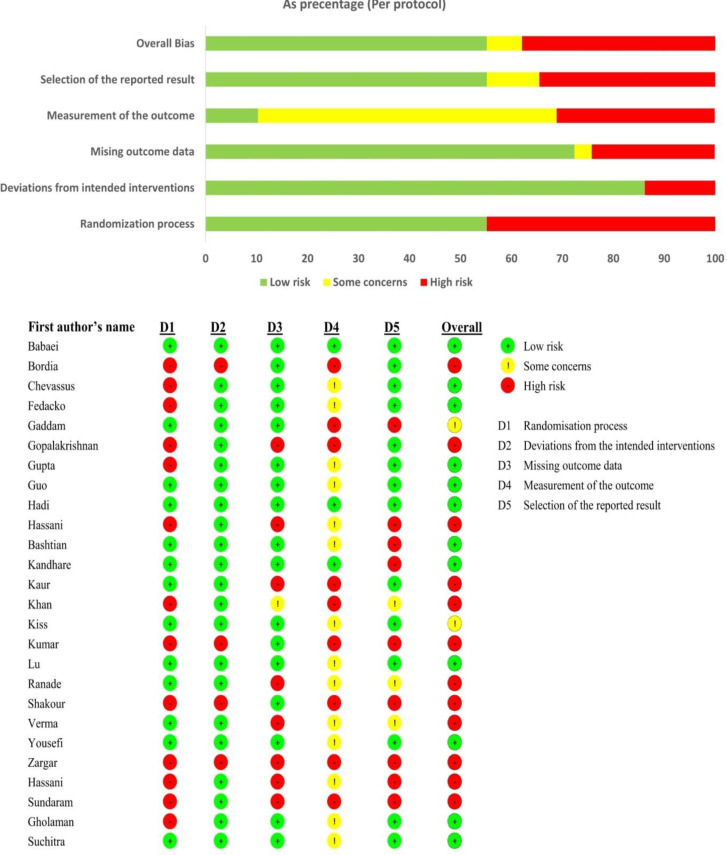
Diagram of risk of bias in the included studies using Cochrane Collaboration’s risk of bias tool 2.

The included papers were assessed for quality using the Risk of Bias Tool 2 (RoB2) (Higgins et al. 2011) developed by the Cochrane Collaboration for RCTs. Two reviewers (LF and FC) assessed the methodological quality separately. The RoB2 tool would take into account the outcome measurement, choice of reported result, missing outcome data, deviations from planned interventions, and randomization process. If the study contained methodological errors, each domain was assigned a "high risk" score; otherwise, it was assigned a "low risk" score; and if there was not enough data to evaluate, it was assigned an "unclear risk" score (Figure 1).

**Statistical analysis**

 The analyses were all performed using STATA-16.0. Changes in FBG, 2hPP, HOMA-IR, HbA1c and Insulin levels were reported as absolute differences between mean values at baseline and end-of-study. Changes between baseline and final standard deviation (SD) values for variables were directly extracted from the articles or calculated assuming a correlation of 0.5 between the baseline and final measures within each group, according to the formula of Follmann et al., (Follmann et al. 1992) [SD(C( =√SD(B)^2^ + SD(F)^2^ − (2 × R × SD(B) × SD(F)) ] as proposed in the Cochrane guidelines (Higgins et al. updated 2023). If the SDs were not available, it was calculated using standard errors (SD= SE × √n), interquartile ranges (Hozo, Djulbegovic and Hozo 2005), or 95% confidence intervals (CI) (SD = √N × (upper limit − lower limit)/3.92). If data were reported as graphs without numerical values, they were converted using Graph Digitizer. Overall estimates of the treatment effect were calculated using a random-effects model and are reported as weighted mean difference (WMD) with 95% CI (DerSimonian and Laird 1986). Since scales used for the assessment of insulin differed across trials, standardized mean differences (SMD) were pooled. The heterogeneity of the study results was estimated by the chi-squared (χ^2^) test and quantified using the I^2^ statistic. Low, moderate, and high heterogeneity were defined as I^2^ index < 25, 50–75, and > 75%, respectively (Higgins et al. 2003). To identify the source of heterogeneity, meta-regression and subgroup analysis were used. Subgroup analyses were performed based on the type of intervention and dose used, duration of intervention, health status, participants' age and baseline BMI. Sensitivity analyses were also performed to assess the influence of each study on the stability of the meta-analysis results. Each time, one study was excluded to show the individual study’s impact on the combined effect estimate. Meta-regression was also used to find sources of heterogeneity and presence of any linear relationships between effect size and study duration, as well as intervention dosage. To calculate the non-linear effects of fenugreek dosage (mg/d) on FBG parameter, fractional polynomial modeling (dose-response analysis) was utilized. Publication bias was assessed visually by funnel plot asymmetry and statistically by Begg’s and Egger’s tests whenever there were greater than 10 studies combined in a meta-analysis. When publication bias was found, trim-and-fills analysis was performed to find out the effect of missed study on the overall effect. A p-value < 0.05 was considered statistically significant. 

## Results

**Figure 2:**
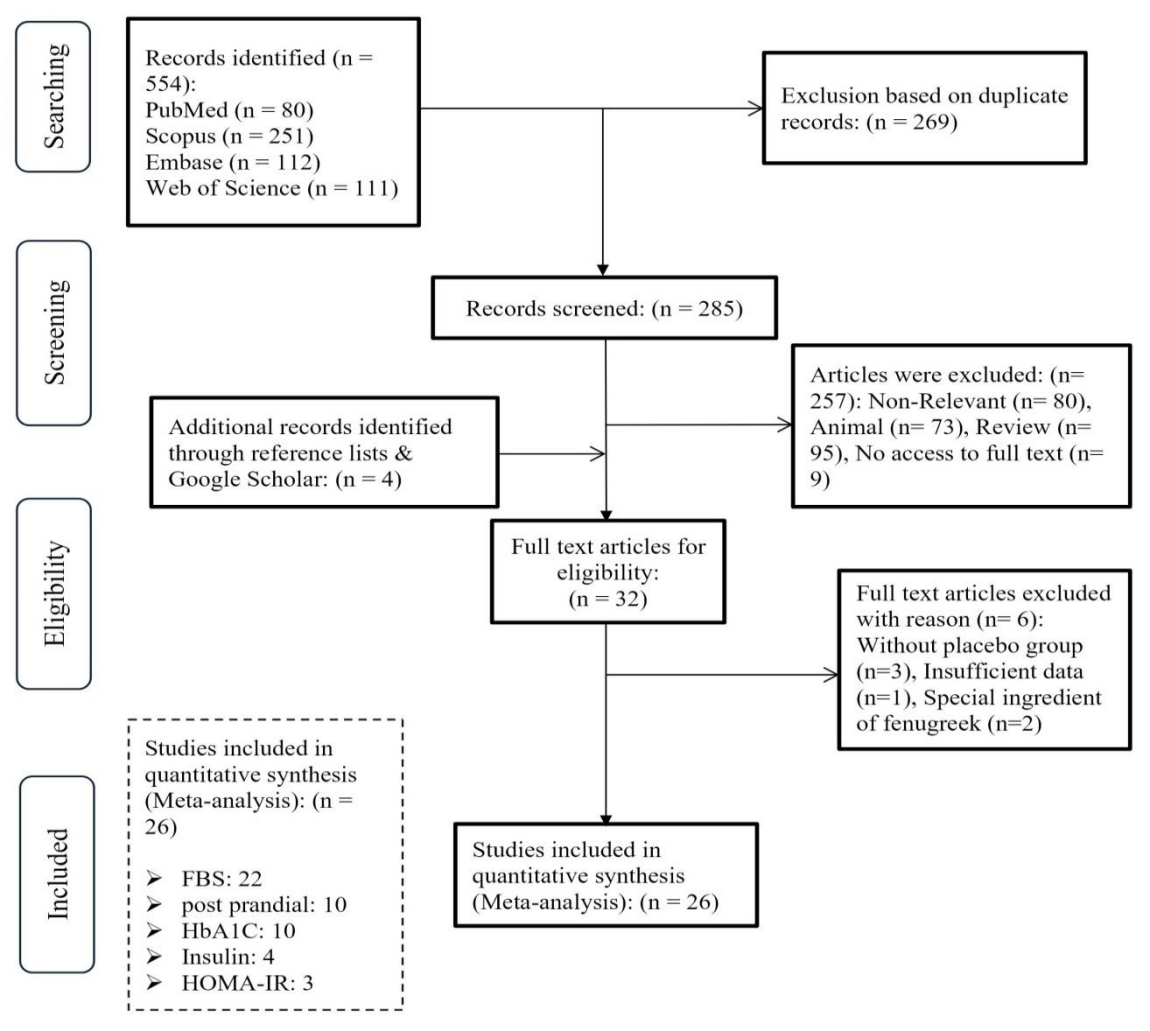
PRISMA 2020 flow diagram of the included studies.

From the initial search strategy through databases, a total of 554 articles were identified (PubMed (n=80), Scopus (n=251), Embase (n=112), and Web of Science (n=111)). After excluding duplicates (n=269), 285 remaining studies were reviewed based on their titles/abstracts by two independent investigators. Of these 285 publications, 28 were selected for full-text assessment. In addition, 4 studies were identified in the references lists of the included studies. After detailed evaluation, 26 RCTs met the inclusion criteria and were selected for meta-analysis. Among these trials, 22 trials with 27 arms assessed FBG (Babaei et al. 2020; Bordia, Verma and Srivastava 1997; Chevassus et al. 2010; Fedacko et al. 2016; Gaddam et al. 2015; Gopalakrishnan et al. 2020; Gupta, Gupta and Lal 2001; Hadi et al. 2020; Hassani et al. 2019a; Hassani et al. 2019b; Hassanzadeh Bashtian et al. 2013; Kamakhya et al. 2015; Kandhare et al. 2018; Kaur 2016; Khan and Khosla 2018; Kiss et al. 2018; Lu et al. 2008; Ranade and Mudgalkar 2017; Shakour et al. 2003; Verma et al. 2016; Yousefi et al. 2017; Zargar et al. 1992), 10 trials with 14 arms reported 2hPPG (Fedacko et al. 2016; Gupta, Gupta and Lal 2001; Kandhare et al. 2018; Kaur 2016; Khan and Khosla 2018; Kooshki et al. 2018; Lu et al. 2008; Shakour et al. 2003; Verma et al. 2016; Zargar et al. 1992), 10 Trials with 11 arms evaluated HbA1C (Gupta, Gupta and Lal 2001; Hassani et al. 2019a; Hassani et al. 2019b; Kamakhya et al. 2015; Kandhare et al. 2018; Lu et al. 2008; Ranade and Mudgalkar 2017; Suchitra and Parthasarathy 2015; Sundaram et al. 2020; Zargar et al. 1992), 4 reported Insulin (Chevassus et al. 2010; Gholaman and Gholami 2018; Hassanzadeh Bashtian et al. 2013; Kiss et al. 2018), and 3 reported HOMA-IR (Gholaman and Gholami 2018; Hassanzadeh Bashtian et al. 2013; Kiss et al. 2018). Reasons for excluding the other 6 full-text articles were as follows: (1) without placebo group (n=3) (Najdi et al. 2019; Sharma and Raghuram 1990; Sowmya and Rajyalakshmi 1999), (2) special ingredient of fenugreek (n=2) (Deshpande et al. 2020; Rashid et al. 2019), (3) and insufficient data (n=1) (Akhtar et al. 2002). Moreover, Shakour et al. used different forms of fenugreek in their study (Shakour et al. 2003), Zargar et al. used different doses (Zargar et al. 1992) and Bordia et al. used different population for their study (Bordia, Verma and Srivastava 1997) that each of which was considered as a separate study in the present study. The PRISMA flow diagram summarizes the results of the study selection process of the present study (Figure 2).

**Systematic review and study characteristics**

**Characteristics of included studies**

The characteristics of the included studies are presented in Table 1. A total of 26 studies involving 833 participants in the intervention group and 792 participants in the control group were included in this systematic review and meta-analysis. The mean age of the participants ranged from 18 to 70 years. The duration of the trials varied from 4 to 144 weeks, and the design of the RCTs was parallel. 

**Meta-analysis results**

**Figure 3:**
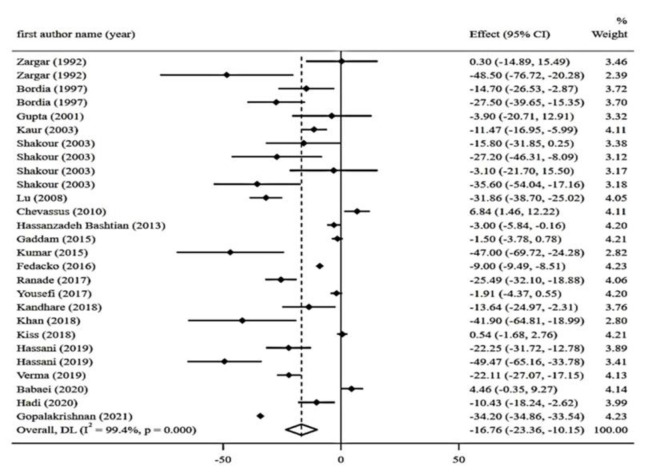
Forest plot reporting weighted mean difference and 95% confidence intervals (CI) for the effects of fenugreek intake on Fasting blood glucose (FBG)

**Figure 4:**
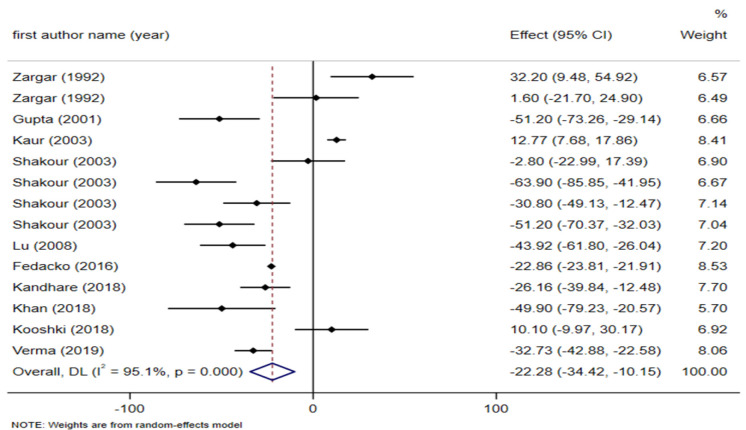
Forest plot reporting weighted mean difference and 95% confidence intervals (CI) for the effects of fenugreek intake on 2-hr postprandial glucose (2hPPG)

Forest plots summarizing the meta-analysis of the trials for the effects of fenugreek supplementation on glycemic parameters including FBG, 2hPPG, HbA1c, HOMA-IR, and Insulin are depicted in Figures 3 to 7.

**Effect of fenugreek on FBG levels**

Twenty-seven arms from 22 RCTs with a total sample size of 1440 individuals (including 743 subjects in the intervention group and 702 controls) have reported FBG. Meta-analysis of studied trials revealed a significant reduction of FBG levels (WMD: - 16.75 mg/dl; 95% CI: - 23.36 to - 10.15; p*<*0.001) with a significant degree of heterogeneity (I^2^ = 99.4%, p*<*0.001) (Figure 3). Subgroup analysis was performed based on mean age, mean BMI, country, health condition, intervention type, dosage, and duration. The results of subgroup analysis revealed that reduction effects of fenugreek on FBG were more significant in BMI≤ 30 kg/m^2^ (BMI: 185-24.9 kg/m^2^; n= 2; WMD -9.12 ml; 95% CI, -9.61 to -8.63; p<0.001; I^2^=97.7%, p<0.001; BMI: 25-29.9 kg/m^2^; n=10; WMD -3.13 ml; 95% CI, -4.28 to -1.99; p<0.001; I^2^=92.4%, p<0.001). Moreover all forms of fenugreek have significant effects on FBS levels p<0.001) (Table 2).

**Table 1:** Characteristics of the included studies

**First author’s name **	**Year **	**N- int **	**Location **	**Mean age (int) **	**Mean BMI (int) **	**Gender **	**Study population **	**Intervention type **	**Dosage (mg/day) **	**Duration (weeks) **	**Outcomes **
Zargar-a	1992	14	Iran	40-60	NR	M/F	T2DM	Seed powder	10000	6	FBG ↔, PPG ↔, HbA1c↔
Zargar-b	1992	14	Iran	40-60	NR	M/F	T2DM	Seed powder	20000	6	FBG↓, PPG↔ HbA1c↔
Bordia-a	1997	20	India	NR	NR	M/F	Severe NIDDM	Seed powder	5000	4	FBG ↔
Bordia-b	1997	20	India	NR	NR	M/F	Mild NIDDM	Seed powder	5000	4	FBG ↓
Gupta	2001	12	India	49.16	26.55	M/F	T2DM	Seed extract	1000	8	FBG ↓, PPG ↓, HbA1c ↓
Shakour-a	2003	20	Egypt	25-45	NR	M/F	T2DM	Boiled seeds	15000	4	FBG ↔, PPG ↔
Shakour-b	2003	20	Egypt	25-45	NR	M/F	T2DM	Germinated Seeds	15000	4	FBG ↔, PPG ↓
Shakour-c	2003	20	Egypt	25-45	NR	M/F	T2DM	Seed powder	15000	4	FBG↔, PPG ↓
Shakour-d	2003	20	Egypt	25-45	NR	M/F	T2DM	Defatted seeds	15000	4	FBG ↔, PPG ↓
Kaur	2003	30	India	52.97	25.17	M/F	T2DM	Seed powder	3000	12	FBG ↓, PPG ↓
Lu	2008	46	China	54.8	24.69	M/F	T2DM	Seed powder	6300	12	FBG ↓, HbA1c ↓,PPG↓,
Chevassus	2010	20	France	18-59	NR	M	Healthy	Seed extract	1176	6	FBG ↔ , insulin↔
Hassanzadeh Bashtian	2013	23	Iran	25	28.67	F	PCOS	Seed extract	1000	8	FBG ↔, HOMA-IR ↔, insulin↔
Gaddam	2015	52	India	30-70	26.62	M/F	T2DM	Seed powder	10000	144	FBG ↓, HbA1c↔
Suchitra	2015	30	India	5.37	NR	M/F	T2DM	Seed	90000	8	HbA1c ↓
Kumar	2015	21	India	40–60	NR	M/F	T2DM	Seed powder	20000	8	FBG ↓, HbA1c↔
Fedacko	2016	29	India	45.8	24.3	M/F	Hyperlipidemia	Defatted seeds	60000	12	FBG ↓, PPG ↓
Ranade	2017	30	India	48	25.23	M/F	T2DM	Seed	10000	24	FBG ↓, HbA1c↔
Yousefi	2017	24	Iran	37.22	27.42	M/F	T2DM	Seed	8000	8	FBG ↔
Gholaman-a	2018	10	Iran	44.2	33.11	F	T2DM	Seed	15000	8	insulin↔, HOMA-IR ↔,
Gholaman-b	2018	10	Iran	44.2	32.56	F	T2DM	Seed	15000	8	insulin↓, HOMA-IR↓,
Kandhare	2018	38	India	51.48	26.08	M/F	T2DM	Seed extract	2100	12	FBG ↓,PPG ↓, HbA1c ↓
Khan	2018	40	India	18–70	31.2	M/F	T2DM	Seed	25000	6.4	FBG ↓, PPG ↓
Kiss	2018	8	Hungary	42.75	27.08	M/F	Healthy	NR	1000	1.4	FBG ↔, Ins ↔, HOMA-IR ↔
Hassani-a	2019	31	Iran	51.23	26.64	M/F	T2DM	Seed powder	10000	8	FBG ↓, HbA1c ↓
Hassani-b	2019	31	Iran	50.19	27.43	M/F	T2DM	Seed powder	10000	8	FBG ↓, HbA1c ↓
Verma	2019	77	India	25-60	NR	M/F	T2DM	Seed extract	1000	12	FBG ↓, PPG ↓
Babaei	2020	13	Iran	39.08	39.08	M/F	NAFLD	Seed extract	1000	12	FBG ↔
Gopalakrishnan	2020	60	India	NR	NR	M/F	T2DM	Seeds extract	NR	4	FBG ↓
Hadi	2020	24	Iran	47.7	30.02	M/F	T2DM	Seed powder	15000	8	FBG ↓
Sundaram	2021	40	India	NR	NR	M/F	T2DM	Seed powder	25	4	FBG ↓, HbA1c↔

Abbreviations: n, number of studies; int, intervention; BMI, body mass index; M, male; F, female; NAFLD, non-alcoholic fatty liver disease; NIDDM, non-insulin-dependent diabetes mellitus; T2DM, type 2 diabetes mellitus; PCOS, polycystic ovary syndrome; FBG, fasting blood glucose; PPG, Postprandial glucose; HbA1c, Hemoglobin A1c; HOMA-IR, Homeostatic Model Assessment for Insulin Resistance ; NR, not reported; ↓ significant reduction; ↑ significant increase; ↔ not changed.

**Table 2:** Subgroup analyses of fenugreek intake on glycemic parameters

**Subgroups**	**Effect size, n**	**WMD (95% CI)**	**p within group**	**I** ^2^	**p heterogeneity**
Subgroup analysis for FBG (mg/dl)					
Overall	28	-16.75 (-23.36, -10.15)	<.001	99.4%	<.001
Dosage (g)					
≤ 1	5	-4.77 (-12.66, 3.11)	.235	94.7%	<.001
1 – 10	12	-15.17 (-22.10, -8.24)	<.001	94.4%	<.001
> 10	10	-25.742 ( -37.93, -13.54)	<.001	99.8%	<.001
Duration (week)					
≤ 4	8	-19.597 (-36.46, -2.73)	.023	99.2%	<.001
4 – 8	11	-14.62 (-21.66, -7.58)	<.001	90.2%	<.001
> 8	8	-13.34 (-19.11, -7.57)	<.001	95.8%	<.001
Health condition					
T2DM	22	-21.37 ( -29.87, -12.86)	<.001	98.5%	<.001
Healthy	2	3.19 (-2.903, 9.29)	.305	77.8%	.034
Other disease	3	-2.86 (-9.81, 4.08)	.419	95.6%	<.001
Intervention type					
Powder	13	-22.28 (-33.70, -10.87)	<.001	98.6%	<.001
Extract	6	-5.03 ( -14.24, 4.18)	.285	93.9%	<.001
Seed	8	-13.60 ( -19.53, -7.67)	<.001	95.0%	<.001
Baseline BMI					
18.5 - 24.9	2	-20.16 (-42.56, 2.23)	.078	97.7%	<.001
25 - 29.9	11	-9.10 (-13.71, -4.50)	<.001	92.2%	<.001
≥ 30	2	-24.24 (-54.85, 6.36)	.121	84.6%	.011
Mean age (year)					
< 45	11	-7.94 (-13.42, -2.45)	.005	91.7%	<.001
≥ 45	13	-17.72 (23.03, -12.42)	<.001	92.6%	<.001
Subgroup analysis for pp (mg/dl)					
Overall	13	-21.85 ( -22.77, -20.94)	.000	95.1%	<.000
Dosage (g)					
≤ 10	6	-1.90 ( -5.88, 2.08)	.349	95.4%	<.000
> 10	7	-22.97 (-23.91, 22.03)	.000	82.2%	<.000
Duration (Higgins et al.)					
< 8	7	-19.15 (-26.68, 11.63)	.000	89.4%	<.479
≥ 8	6	-21.89 ( -22.82, -20.97)	.000	97.5%	<.479
Intervention type					
Powder	5	6.95 (2.52, 11.38)	.000	91.3%	<.000
Extract	3	-32.92 (-40.54, -25.25)	.167	44.1%	<.000
Seed	5	-22.99 (-23.93, -22.05)	.000	86.1%	<.000
Mean age (year)					
< 45	6	-35.37 ( -42.26, -28.49)	.001	75.7%	<.000
≥ 45	7	-21.61 (-22.53, -20.69)	.000	97%	<.000
Subgroup analysis for HOMA-IR					
Overall	3	-0.41 (-0.84, 0.02)	<.838	0.0%	<.001
Subgroup analysis for Insulin					
Overall	4	-0.42 (-0.79, -0.05)	.226	31.1%	<.001

Abbreviations: n; number of studies; WMD, weighted mean differences; CI, confidence interval; FBG, fasting blood glucose; T2DM, type 2 diabetes mellitus; Postprandial glucose; HbA1c, Hemoglobin A1c; HOMA-IR, Homeostatic Model Assessment for Insulin Resistance

**Effect of fenugreek on 2 hours post prandial glucose (2hPP)**

Analysis of 10 trials with 14 arms for post prandial glucose with a total of 840 participants (including 406 subjects in the intervention group and 434 controls) demonstrated that fenugreek intake had a significant effect on decreasing 2hPP (WMD: - 22.28 mg/dl; 95% CI: - 34.42 to - 10.15; p<0.001; I² (%): 95.1%, p<0.001) (Figure 4). Subgroup analysis revealed that intervention with seed (WMD: - 22.99 mg/dl; 95% CI: - 23.93 to - 22.05; p<0.001; I² (%): 86.1%, p<0.001) and extract (WMD: - 32.90 mg/dl; 95 % CI: - 40.54 to - 25.25; p<0.001; I² (%): 44.1%, p = 0.167) types of fenugreek and dosages of > 10 g (WMD: - 22.97 mg/dl; 95% CI: - 23.91 to - 22.03; p<0.001; I² (%): 82.2%, p<0.001) showed a substantial and potent decreasing effect on postprandial glucose compared to other subgroups (Table 2).

**Effect of fenugreek on HbA1c levels**

In total, ten RCTs with 11 arms and a total sample size of 787 individuals (including 307 subjects in the intervention group and 480 controls) have reported HbA1c levels and were included in the meta-analysis. The results of the pooled analysis showed a significant reduction in HbA1c levels (WMD: - 0.63 mg/dl; 95% CI: - 0.75 to - 0.51; p*<*0.001) with a significant degree of heterogeneity (I2 = 47.3%, p = 0.041) (Figure 5.1). Regarding subgroup analysis, powder types of fenugreek (WMD: - 0.74 mg/dl; 95% CI: - 0.90 to - 0.58; p*<*0.001; I^2^=57.2%, p = 0.029) and extract types of fenugreek (WMD: - 0.48 mg/dl; 95% CI: - 0.69 to - 0.28; p*<*0.001; I^2^=0.0%, p= 0.553) (Figure 5.2), intervention dosage with ≥10 g/day (WMD: - 0.42 mg/dl; 95% CI: - 0.64 to - 0.20; p*<*0.001; I^2^=0.0%, p = 0.987) (Figure 5.3) and durations ≥ 8 weeks (WMD: - 0.54 mg/dl; 95% CI: - 0.69 to - 0.40; p*<*0.001; I^2^=4.1%, p= 0.399) (Figure 5.4), had a significant and notable lowering impact on HbA1c levels compared to other subgroups and as showed, the heterogeneity has decreased significantly in these subgroups. 

**Figure 5-1:**
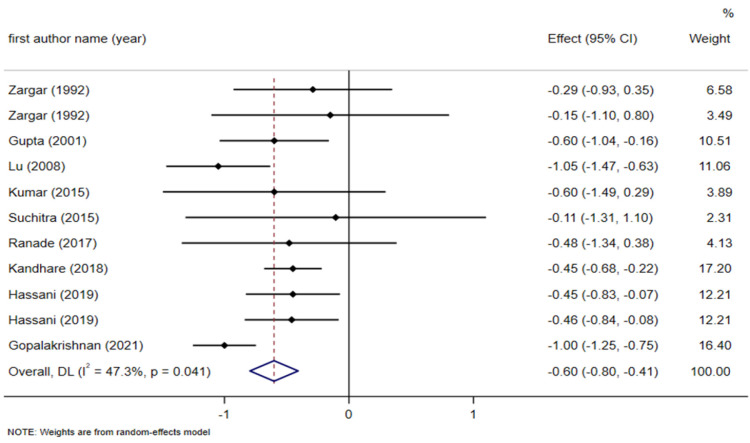
Forest plot reporting weighted mean difference and 95% confidence intervals (CI) for the effects of fenugreek intake on Hemoglobin A1C (HbA1C)

**Figure 5-2:**
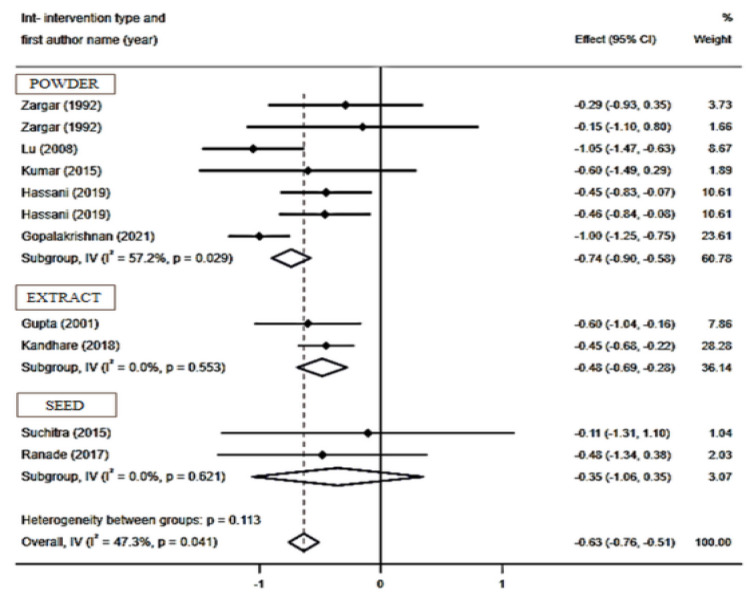
Forest plot reporting weighted mean difference and 95% confidence intervals (CI) for the effects of fenugreek intake on Hemoglobin A1C (HbA1C) based on intervention type

**Figure 5-3:**
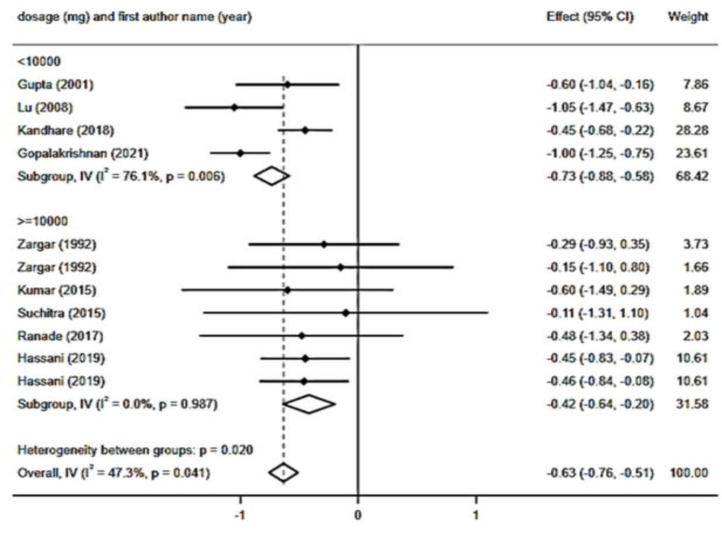
Forest plot reporting weighted mean difference and 95% confidence intervals (CI) for the effects of fenugreek intake on Hemoglobin A1C (HbA1C) based on intervention dosage

**Figure 5-4:**
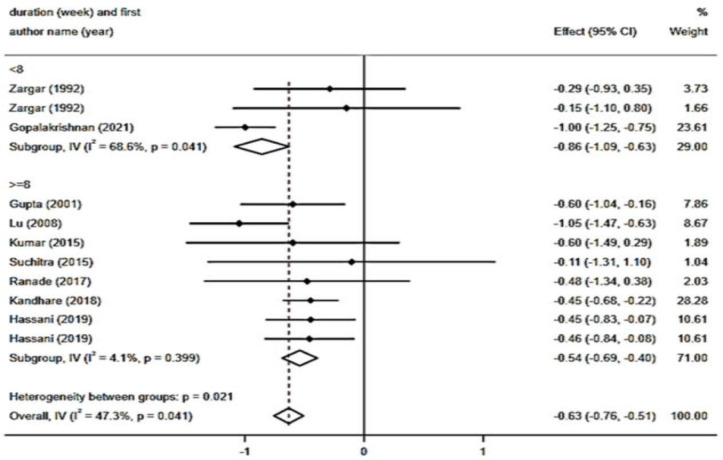
Forest plot reporting weighted mean difference and 95% confidence intervals (CI) for the effects of fenugreek intake on Hemoglobin A1C (HbA1C) based on intervention duration

**Effect of fenugreek on insulin**

**Figure 6:**
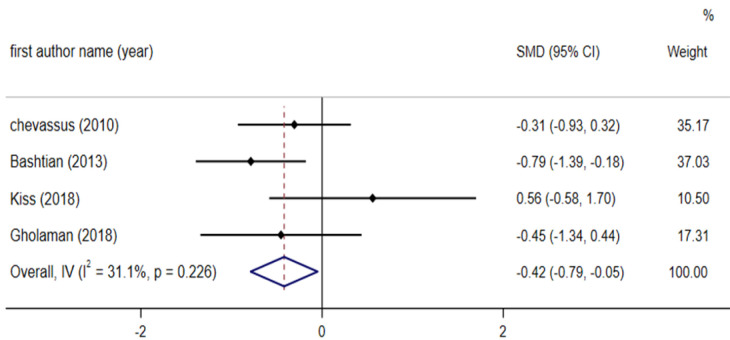
Forest plot reporting weighted mean difference and 95% confidence intervals (CI) for the effects of fenugreek intake on Insulin

Combined results of four studies with a total of 118 participants (including 61 subjects in the intervention group and 57 controls), showed that fenugreek consumption significantlydecreased insulin (SMD: - 0.42; 95% CI: - 0.79 to - 0.05; p = 0.026), without significant heterogeneity among the studies (I^2^ = 31.1%, p = 0.226) (Figure 6).

**Effect of fenugreek on HOMA-IR**

**Figure 7:**
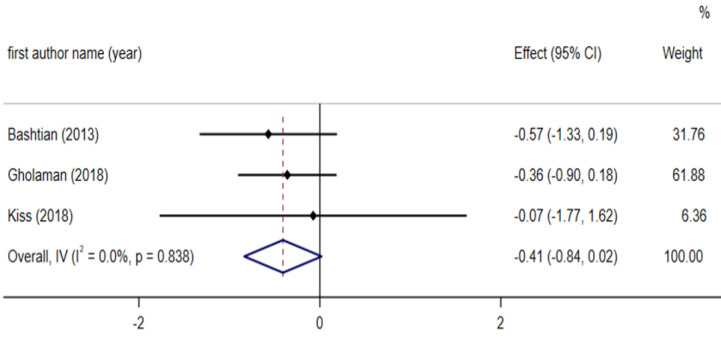
Forest plot reporting weighted mean difference and 95% confidence intervals (CI) for the effects of fenugreek intake on Insulin resistance (HOMA-IR)

Pooled results of the random-effect model on three studies showed that fenugreek intake did not affect HOMA-IR significantly (WMD: -22.28 mg/dl; 95% CI: - 0.84 to 0.02; p = 0.061) (Figure 7). The heterogeneity across the studies was not significant (I^2^ = 0.0 %, p = 0.838). 

**Meta-regression and non-linear dose** **response analysis **

**Figure 8:**
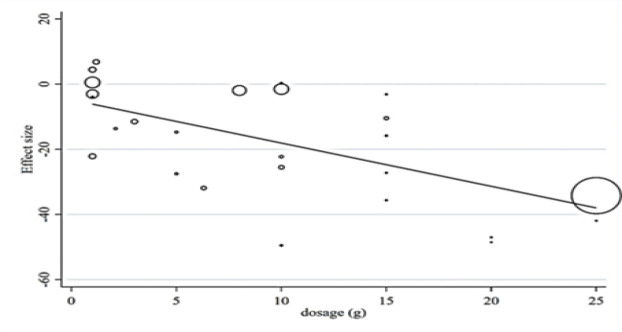
Meta-regression analysis between fenugreek dosage and mean difference in (A) Fasting blood glucose (FBG)

**Figure 9:**
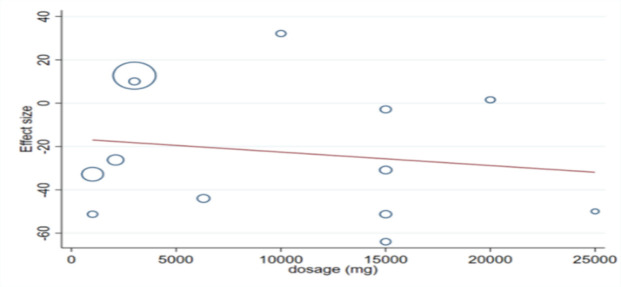
Meta-regression analysis between fenugreek dosage and mean difference in (B) 2-hr postprandial glucose (2hPPG)

**Figure 10:**
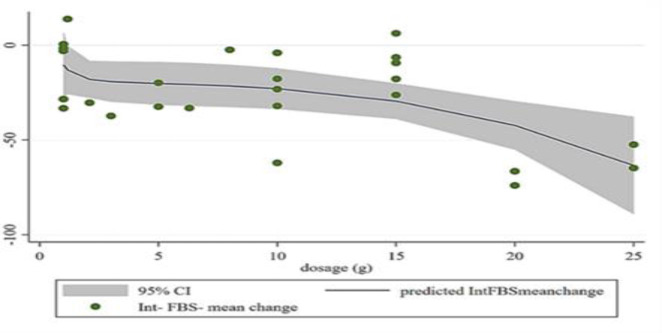
Non-linear dose-response analysis between fenugreek dosage and mean difference in (C) Fasting blood glucose (FBG)

The results of random-effect meta regression showed that after removing two studies (Fedacko et al. 2016; Suchitra and Parthasarathy 2015), due to the large dosage difference among other studies (administration of 60 and 90 g fenugreek respectively), there was a significant and negative linear relationship between the dose of fenugreek (g/day) and absolute changes in FBG (Coef. =- 3.64, plinear < 0.001) and 2hPP (Coef. = - 0.54, plinear < 0.001) (Figures 8 to 9). Also, non-linear dose-response analysis illustrated a significant association between fenugreek dosage and FBG (Coef. = − 2.86, pnon-linearity = 0.009). It seems that the highest reducing effect for FBG occurs at the dosages of 15 g/day fenugreek (Figure 10).

**Sensitivity analysis**

**Figure 11:**
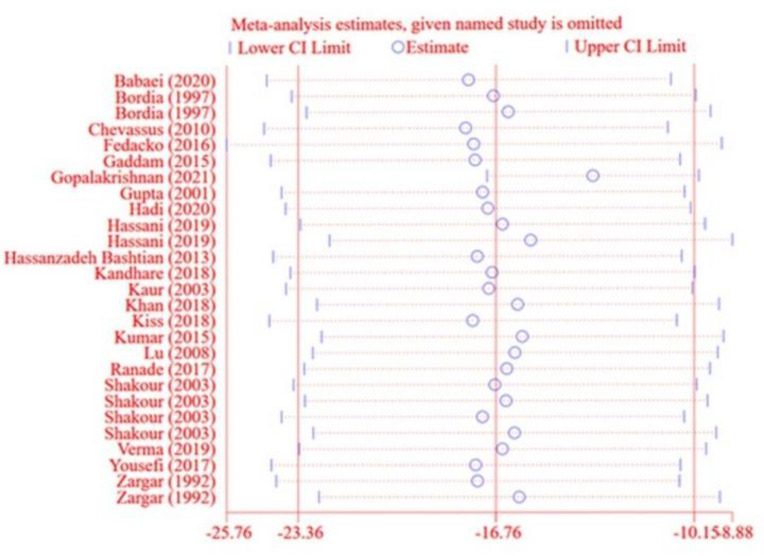
Sensitivity analysis for Fasting blood glucose (FBG)

**Figure 12:**
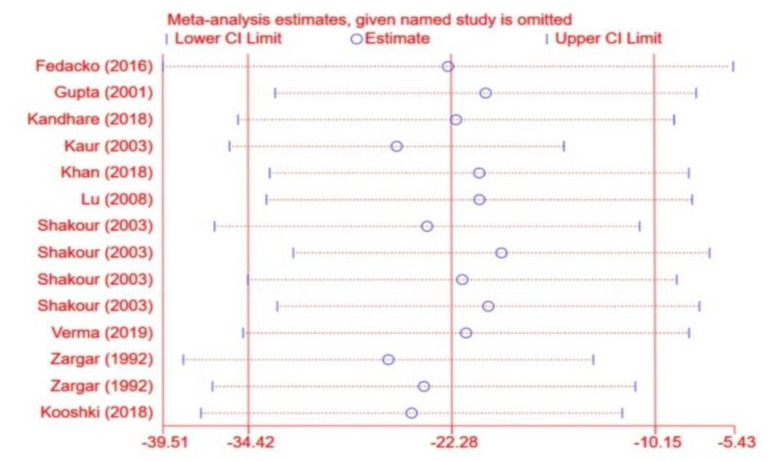
Sensitivity analysis for 2h postprandial glucose (2hPPG)

To determine the inﬂuence of each effect size on the overall effect size, every study was excluded from the analysis one by one. We found no significant effect of any single study on the overall effect sizes of FBG, and 2hPPG (Figures 11 to 12).

**Publication bias**

**Figure 13:**
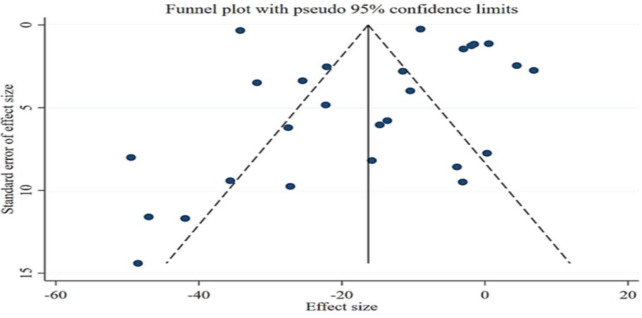
Funnel plot representing publication bias for the impact of fenugreek intake on Fasting blood glucose (FBG)

**Figure 14:**
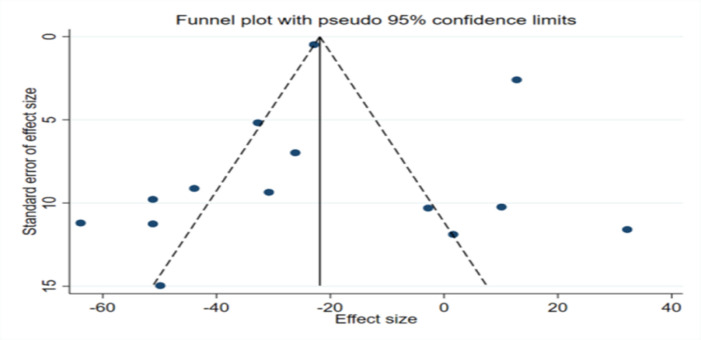
Funnel plot representing publication bias for the impact of fenugreek intake on 2-hr postprandial glucose (2hPPG)

**Figure 15:**
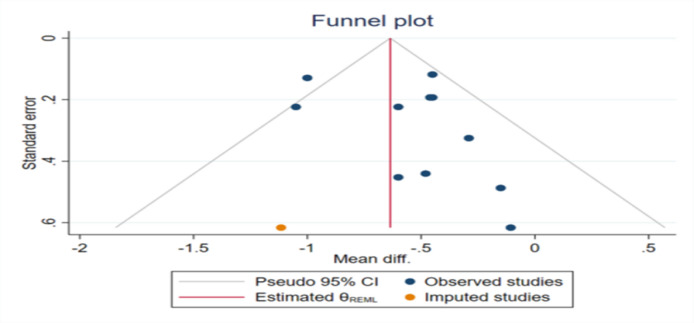
Funnel plot representing publication bias for the impact of fenugreek intake on Hemoglobin A1C (HbA1C)

The results of Egger’s and Begg’s tests for FBG and post prandial glucose did not support the existence of publication bias. However, the funnel plot for HbA1c and was similarly asymmetric (Figure 13 to 15). Therefore, trim-and-fill analysis was applied to find out probably missed studies. There was one imputed study for HbA1c and the effect size changed as (WMD: -0.63; 95% CI: -0.76 to -0.51; p<0.001).

## Discussion

A comprehensive evaluation of the impact of fenugreek consumption on glycemic parameters, such as FBG, 2hPP, HbA1c, insulin levels, and HOMA-IR, was conducted for the first time in the current meta-analysis of 26 trials. The present study demonstrated that fenugreek significantly reduced FBG by 16.76 mg/dl, 2hPP by 22.28 mg/dl, HbA1c by 0.63%, and insulin by 0.42 mU/ml. However, it failed to improve HOMA‑IR significantly and we found no evidence of significant heterogeneity for HOMA‑IR and Insulin while a significant heterogeneity was observed for FBG, 2hPP, and HbA1c among the included trials. Discrepancies in daily fenugreek dosage and intervention duration, type of fenugreek used, and BMI and age of participants could have influenced this heterogeneity. The results of the subgroup analysis revealed that the effects of fenugreek on FBG reduction were more significant in individuals with a BMI of ≤ 30 kg/m², although this did not significantly decrease heterogeneity. Moreover all forms of fenugreek have significant effects on FBS levels. Additionally, both powder and extract forms of fenugreek, as well as intervention dosages of ≥10 g/day and durations of ≥8 weeks, had a significant and notable impact on lowering HbA1c levels and significantly decreased heterogeneity. In addition, intervention with both seed and extract forms of fenugreek at dosages greater than 10 g/day, although could not significantly decrease heterogeneity, had a substantial and potent effect on reducing 2hPP. Furthermore, the random-effects meta-regression confirmed that, after removing two studies (Fedacko et al. 2016; Suchitra and Parthasarathy 2015) due to significant dosage differences compared to the others, there was a significant negative linear relationship between doses of fenugreek (g/day) and absolute changes in FBG and 2hPP. Additionally, the non-linear dose-response analysis illustrated a significant association between fenugreek dosage and FBG, indicating that the greatest reduction in FBG occurs at a dosage of 15 g/day. Moreover, the sensitivity analysis found no significant effect of any single study on the overall effect sizes for FBG and 2hPP. Other potential sources of heterogeneity may include the dietary habits of the participants, the type and dosage of anti-diabetic medications taken, the duration of diabetes, and the physical activity levels of the study participants. These factors were not considered in many trials. Additionally, the results may have been influenced by variations in the quality and purity of the composition. This study is the first comprehensive meta-analysis about the effects of fenugreek on glycemic parameters, especially Insulin, and considered many more studies including 26 trials compared to the 10 to 12 trials included in previous meta-analyses (Khodamoradi et al. 2020; Kim et al. 2023b; Shabil et al. 2023). In some cases, the findings of our study were inconsistent. In contrast, in some others, they aligned with those of previous systematic reviews and meta-analyses regarding the effects of fenugreek consumption on glycemic parameters. A systematic review with 14 trials and a total of 894 participants, focusing on the effects of fenugreek on outcomes such as FBG, 2hPP, and HbA1c, revealed a non-significant reduction in FBG and 2hPP, while a significant reduction in HbA1c was observed. The authors noted substantial heterogeneity among the studies, attributed to variations in diabetes status, fenugreek dosage, and study quality (Shabil et al. 2023). The number of studies (26 trials) and the larger sample sizes (833 participants in the intervention group and 792 participants in the control group) assessed in our study increased the statistical power of the results compared to the aforementioned study. This may be a possible reason for the inconsistent results observed regarding FBG and 2hPP. In another meta-analysis of 10 articles (12 studies with a total sample size of 1173) published in 2016, Gong et al. investigated the efficacy of fenugreek (*Trigonella foenum-graecum*) in managing hyperglycemia in individuals with diabetes mellitus and prediabetes. The analysis showed a significant decline in FBG and HbA1c. However, the study did not investigate insulin levels or HOMA-IR (Gong et al. 2016). Our results were in line with a recent meta-analysis in 2023 (Kim et al. 2023a) that investigated the impact of fenugreek (*Trigonella foenum-graecum*) on glycemic control in individuals with T2DM and prediabetes. A total of 10 studies involving 706 participants reported a significant reduction in FBG, 2hPP, and HbA1c, without significant effects on HOMA-IR. However, changes in insulin levels were not considered in this study, and no subgroup analysis was conducted to examine the impact of various factors on the results. In our study, by conducting a subgroup analysis, it was determined that the intervention did not effectively reduce FBG levels in healthy participants which was aligned with the results of Neelakantan et al. with a total of 10 trials and a sample size of 278, which reported the beneficial effects of medium or high doses of fenugreek on FBG and 2hPP only in persons with diabetes. (Neelakantan et al. 2014). 

Additionally, a review study on the hypoglycemic effects of certain herbal foods concluded that, based on the quality of trials and available evidence, consuming fenugreek could lower FBS levels in diabetic patients, but it was not effective in healthy, overweight, and obese individuals (Deng 2012). Based on the trial conducted by Mathern et al., it was reported that fenugreek could potentially influence blood glucose levels in response to higher amounts of glucose. Consequently, fenugreek consumption may be more beneficial for individuals with T2DM who have disrupted glucose metabolism and elevated blood glucose levels (Mathern et al. 2009). Hence, insufficient trials involving healthy individuals and the higher baseline blood glucose levels in diabetic patients compared to healthy subjects could help clarify our observations in this specific group.

Limited studies have investigated the effect of fenugreek on insulin levels and HOMA-IR, finding non-significant reductions in these parameters. However, the estimated effect size of the pooled results from four trials reporting the effect of fenugreek on insulin levels (Chevassus et al. 2010; Gholaman and Gholami 2018; Hassanzadeh Bashtian et al. 2013; Kiss et al. 2018), and three trials reporting on HOMA-IR (Gholaman and Gholami 2018; Hassanzadeh Bashtian et al. 2013; Kiss et al. 2018), indicated a significant reduction in insulin levels in the intervention groups compared with placebo. This reduction may partly be attributed to fenugreek's lowering effects on blood glucose levels and suggests an increase in insulin sensitivity, along with a decreased need for additional insulin secretion. Although HOMA-IR was also reduced, this change was not statistically significant. The insufficient power due to low sample sizes in the original trials, as well as the absence of hyperinsulinemia in all subjects at the beginning of the study, likely accounted for the differing conclusions obtained in these trials. 

The most researched portion of the fenugreek plant is the seeds, which contain the following components: 30% galactomannan, 20% insoluble fiber, 20–30% protein (primarily in the form of 4-OH-Ile at 80%), 5–10% fat, and alkaloids such as trigonelline and steroidal saponins, including diosgenin. The diverse components of fenugreek suggest that there are various mechanisms underlying its health benefits (Syed et al. 2020; Yao et al. 2020). The soluble dietary fiber found in fenugreek seeds, known as galactomannan, is the most abundant component and influences the glycemic response by inhibiting and delaying the action of digestive and absorptive enzymes, improving gastrointestinal motility, and balancing the microbiome. By protecting islet cells, reducing insulin resistance, and ultimately enhancing glucose absorption, galactomannan promotes insulin production (Deshpande et al. 2020; Fuller and Stephens 2015). Moreover, 4-OH-Ile has shown effects on insulin-sensitizing and insulin-secreting hepatic cells and peripheral organs (Yao et al. 2020) . Furthermore, by directly targeting white adipose tissue, diosgenin has both an insulin-secreting and sensitizing effect by lowering beta cell oxidative stress, restoring their function, and ultimately enhancing peripheral tissue glucose uptake. (Fuller and Stephens 2015; Saravanan et al. 2014; Yao et al. 2020).

To the best of our knowledge, the current review is the first comprehensive meta-analysis about the effects of fenugreek on glycemic parameters, especially insulin, and considered many more studies including 26 trials in comparison to the 10 to 12 trials included in previous meta-analyses. However, our study has some limitations. Our search was limited to English-language publications. Therefore, articles written in other languages were not used. In addition, according to the poor quality of some articles that were included, significant heterogeneity was observed in the data. Furthermore, we were unable to identify any potential sources of heterogeneity for the FBG variable. Additionally, including patients with various metabolic conditions in the study could limit the generalizability of the results. Moreover, subgroup analysis was not performed for insulin and HOMA-IR due to the limited number of studies. 

Overall, fenugreek supplementation significantly improved FBG, 2hPP, HbA1c, and insulin but did not affect HOMA-IR. The present study supports the clinical application of fenugreek for patients with diabetes, however, further RCTs of high methodological quality with adequate sample sizes are required to expand our findings, validate fenugreek's efficacy as a hyperglycemic remedy, and explore its long-term eﬀects.

## Data Availability

Data will be made available on request.
